# Working from anywhere: yin–yang cognition paradoxes of knowledge sharing and hiding for developing careers in China

**DOI:** 10.1057/s41599-023-01744-5

**Published:** 2023-05-15

**Authors:** Tachia Chin, Yi Shi, Manlio Del Giudice, Jianwei Meng, Zeyu Xing

**Affiliations:** 1 School of Management, Zhejiang University of Technology, Hangzhou, China; 2 University of Rome “Link Campus University”, Rome, Italy; 3 School of Marxism Studies, University of Chinese Academy of Sciences, Beijing, China

**Keywords:** Business and management, Cultural and media studies, Business and management

## Abstract

Digital technology coupled with the quarantines caused by the COVID-19 pandemic has made working from anywhere (WFA)—a modern form of remote working—a widespread phenomenon. Given that WFA brings new career challenges to and engenders paradoxes of knowledge exchange among employees, this research aims to examine how the interactions of remote work time (RWT), knowledge sharing (KS), and knowledge hiding (KH) affect career development (CD) from a culturally grounded paradoxical framing of yin–yang harmonizing. The data were collected from Chinese manufacturing employees, and a moderated hierarchical regression analysis was used to examine the hypotheses. The results show an inverted U-shaped relationship between RWT and CD. The interaction of KS and KH is significantly related to CD, and the inverted U-shaped RWT–CD relationship is moderated by the interaction term, in which RWT exerts the most substantial positive impact on CD when KS is high and KH is low. This study offers valuable implications for coping with perplexing employment relationships and increasing career challenges in volatile work environments. The primary originality is to adopt a novel cognitive frame of yin–yang harmonizing to examine the nonlinear effect of remote working and the symbiotic impact of KS and KH on CD, which not only enriches the understanding of flexible work arrangements in the digital economy but also provides novel insights into the interconnectedness of KS and KH and their interacting effects on HRM-related outcomes.

## Introduction

The rapid advancement in information and communication technology (ICT), coupled with the mandated quarantines caused by the COVID-19 pandemic, has compelled numerous employees across the globe to engage in part- or full-time remote work. Thus, working from anywhere (WFA; Bloom, 2014)—a digital-enabled, unconventional form of working requiring flexible yet convoluted job arrangements (e.g., a mix of online and remote work) and agile human resources management (HRM) systems—has been gaining momentum. However, although the Fourth Industrial Revolution made telework and home-based working prevalent for years (DeFilippis et al., 2022; Kwon and Jeon, 2020), many companies, even tech giants like Microsoft, Twitter, and Facebook, did not allow employees to work from home indefinitely and on a large scale, as seen in the modern WFA mode emerging from the pandemic.

Although the implementation of hybrid work models consisting of remote and office work is a nascent phenomenon, it is becoming more commonplace and may fundamentally alter the career landscape of global workers in the near future. However, only a few studies have dived into the impact of WFA on career-related outcomes, of which positive and negative relationships have been reported (Moens et al., 2022; Toniolo-Barrios and Pitt, 2020). On the one hand, WFA might enhance employees’ career prospects by increasing their autonomy and intrinsic motivation (Choudhury et al., 2019); on the other hand, it could decrease employees’ efforts to cling to their jobs because of the lowering of organizations’ control and monitoring (Rupietta and Beckmann, 2018). In light of these controversial findings, a deeper, more comprehensive investigation is urgently required. Thus, we aim to address this lacuna in the present study.

Microsoft’s company-wide WFA policies during the COVID-19 pandemic can be treated as a valuable natural experiment^1^. According to recent empirical surveys by Microsoft (Rodeghero et al. 2021; Yang et al. 2021), firm-wide remote work allowed for more multitasking that could facilitate the sharing of diverse knowledge in parallel. However, it also reduced the quality of the workers’ output because the transfer of knowledge was largely impeded by a decrease in synchronous communication and social connections among team members. These facts bring to light the complexity of knowledge exchange in the WFA setting, where a lack of physical embodiment may hinder learning and communication (Danish et al., 2020; Hardy, 2020; Wallace, 2020). Specifically, in WFA practices, ICT is instrumental in knowledge sharing (KS) between remote and on-site employees (Donnelly and Johns, 2020; Papa et al., 2020; Saura et al., 2022). Nevertheless, when face-to-face interactions and a shared physical environment are largely replaced by emails, ICT-enabled virtual meetings, and instant communication media, ineluctable distancing limitations may also result in knowledge hiding (KH) among colleagues.

The crucial case of Microsoft draws our attention to the coexistence and seemingly paradoxical roles of KS and KH in the nontraditional WFA context because it implies the synchronous interactions of KS and KH when applying the WFA policy or a hybrid job design comprising remote and on-site work. According to the literature, KS is the actions by individuals to allow knowledge to be available to colleagues (Hooff and Ridder, 2004), and KH is the actions by individuals to conceal the knowledge requested by colleagues (Connelly et al., 2012). Following this logic, investigating the synergies of KS and KH may help capture the changing dynamics of knowledge exchange in a unique WFA context. It appears especially meaningful to explore relevant issues through a novel paradoxical lens that advocates for a persistent symbiosis of contradictory yet interrelated elements, such as KS and KH, over time (Fairhurst et al., 2016).

A counterpart of Western paradoxical theorizing is the cognitive frame of yin–yang harmonizing (Redding, 2017). Given that it can be adopted as an open system to encode a wide range of complementary contradictions, such as the yin and yang elements of a whole at micro levels (Chin et al., 2018, 2021c), the present research uses yin–yang cognition to map the paradoxes. Taking the discussions above together, while previous studies have shown that WFA may largely affect employees’ career development (CD) in the contemporary digital economy (Moens et al., 2022; Toniolo-Barrios and Pitt, 2020), the primary purpose of the current research is to explore the following questions:

1. *How is WFA related to CD among employees?*
2. *How does the interaction of KS and KH affect the relationship between WFA and CD?*

The main contributions of the present paper are threefold. First, we contribute to the HRM literature by employing an unconventional paradoxical approach (i.e., yin–yang cognition) to address the tensions of KS and KH in the ICT-enabled WFA context, in which HRM systems must syncretize office, remote, and a variety of mixed job designs. This advances the traditional binary analysis of on-site and online work to a new, contemporary understanding of the dynamics of work arrangements in the digital economy. Related to this point, our research also adds value to the knowledge management (KM) literature because we provide novel insights into the interconnectedness and synergic mechanisms of employee KS and KH in HRM-related outcomes. Practically, our findings offer valuable implications for global managers and workers in coping with perplexing employment relationships and increasing career challenges in a digitalized, volatile work environment.

The present paper is structured as follows: The section “Theoretical foundation” provides the theoretical foundations for yin–yang harmonizing, paradox framing, and KS and KH in CD. Section “Methodology” describes the methodology used, section “Results” explores the results, and section “Discussion” provides the conclusion, managerial implications, limitations, and future research directions.

## Theoretical foundation

### The cognitive methodology of yin–yang harmonizing as a paradoxical framework

The philosophical origins of *paradoxical framing* are deeply rooted in ancient Western and Eastern cultures, as characterized by Socrates’ dialectical approach and Chinese yin–yang harmonizing dialectics (Lai, 2008). Yin–yang is recognized as the counterpart of the paradox theory that prevails in Western countries (Smith and Lewis, 2011). The only difference between Eastern yin–yang harmonizing and Western paradoxical framing seems to be how these concepts are expressed (Chin et al., 2021a, 2021b, 2021c). For example, the former prefers the application of the yin–yang metaphor as an “open system” to cognitively map all types of paradoxes. As shown in Chinese medical scriptures, the *Medical Classic of the Yellow Emperor* (黄帝内经) and *Compendium and Materia Medica* (本草纲目), yin and yang elements were used as metaphors for Chinese herbs and human organs to decipher their attributes and prescribe treatments for disease. In contrast, the latter favors the use of different metonymic and metaphorical models to map different paradoxes.

Except for the difference described above, the dialectic essences of yin–yang harmonizing and paradox are mutually inclusive; both refer to *a persistent symbiosis of multiple opposing yet interdependent elements* that can be adopted as a cognitive approach to social science sense-making (Keller et al., 2017; Schad et al., 2016). Their underlying logic embraces the existence of two conflicting yet complementary elements, processes, and aspects of a whole in everything (e.g., yin and yang of a thing, day and night, and the good and evil sides of human nature). These seemly opposite dualities are not only conflicting as trade-offs, but also complementary as synergy (Chin et al., 2021a, 2021b, 2021c).

However, yin often represents soft power, passive energies, and feminine aspects, whereas yang represents brutal strength, positive force, and masculine aspects (Lai, 2008; Zhou, 2004). Using the symbiosis interactions of yin and yang shown in the Taiji symbol as an exemplification: conflicting as a trade-off shows that yin and yang wane and wax between each other. The waning of yang leads to the waxing of yin and vice versa, whereas complementary synergy indicates that yin and yang complement each other and are mutually reinforcing. In sum, yin and yang signify an ever-changing, transitional equilibrium rather than a static state as the curvy line that halves the circle describes constant balancing actions (Lloyd, 1996). Following this logic, in any open system, the gray areas between the two opposite poles can be described and captured by the dynamic processes of yin–yang syncretism.

Scholars have also indicated that yin–yang harmonizing cognition is pervasively used by Eastern individuals to interpret the dynamic, contradictory, yet complementary nature of everything (Chin et al., 2021c), while only some Western intellectuals make sense of the causation of opposing categories through a paradoxical lens (Keller et al., 2017). Because the cognitive frame of yin–yang harmonizing has been employed to encode paradoxical mechanisms at the micro level (Chin et al., 2021c; Redding, 2017), we adopt it as our theoretical underpinning to develop the hypotheses.

### The impact of remote work on career development: Yin–yang cognition

As a primary constituent of human resource development (HRD), CD refers to “a mechanism of ensuring the match between the needs of both individual and the organization while developing individuals’ capacity to exercise control over the quality and nature of their career conditions” (Yoon et al., 2021, p. 205). At first glance, the term CD seems to have some overlap with the notion of career sustainability (Chin et al., 2021a; Van der Heijden and De Vos, 2015), as both reflect the evolving sequence of an individual’s different career experiences across time and social spaces. However, their conceptual connotations are somewhat different: CD emphasizes the importance of individuals’ control over their career transitions at different time points (Autin et al., 2020), while career sustainability underscores the meaning, self-realization, and well-being provided by sequences of career experiences over various time periods to individuals (Bal et al., 2020; Chin et al., 2021a). Given that our research was conducted at a specific time point in the postpandemic period, we focus on CD.

Recent research has highlighted that the COVID-19 pandemic gave rise to unexpected layoffs, wage cuts, sudden furloughs, and additional unstable periods of remote working (Autin et al., 2020; Yoon et al., 2021), which not only decreased productivity and well-being but also resulted in life-altering employment shifts all over the globe. Considering that the implementation of WFA has become a necessity for contemporary organizations, it is of particular significance to examine how WFA influences employee CD in the postpandemic world.

When organizations were compelled to apply a highly flexible job design encompassing remote and on-site work to mandatory hotel or home quarantines during the pandemic, the originally stable career statuses and cycles of employees were interrupted by these sudden, unexpected changes in work settings and job characteristics. As a result, the most prominent feature of the modern WFA model lies in its hybridity of the proportion of remote working time to total working time.

The impacts on CD of working remotely have been discussed in the literature but remain ambiguous (Moens et al., 2022; Toniolo-Barrios and Pitt, 2020). Some scholars have suggested that remote work could benefit employees’ CD because it enhances employees’ intrinsic motivation and effort by increasing the degree to which a job offers autonomy and discretion to the individual (Rupietta and Beckmann, 2018). Nevertheless, ironically, evidence also indicates that, in the absence of supervisors as job monitors and colleagues as learning partners on-site (i.e., the lack of social contacts and face-to-face communication), employees may also feel isolated and demotivated when working remotely for too long and too frequently (Donnelly and Johns, 2020).

Focusing on the self-motivational paradoxes about remote work, we use the cognitive methodology of yin–yang harmonizing to frame the relationship between the newly emerged hybrid WFA model consisting of remote and on-site work and CD within a particular condition: the postpandemic era. Specifically, in a mixed job design, the ever-changing proportion of remote work time (RWT) indicates the coexistence of two opposing cognitions as yin and yang elements of a whole that could enable individuals to develop or inhibit their careers, depending on which element is dominant. In other words, RWT can be regarded as a motivator or demotivator for individuals to achieve the set goals of their careers.

The impromptu, ever-changing restrictions caused by the evolving pandemic have detached numerous workers from traditional fixed places of work. In the beginning, workers may feel very motivated when they perceive greater flexibility and autonomy in terms of work time and space. However, after a certain period, individuals may also feel demotivated because they are disassociated with and disengaged from their previous social relationships constructed in the office setting. This implies that, when RWT is lower or higher, individuals are very likely to be affected by initial disruptions or the prolonged effects of remote work fatigue. Following this logic, we assume a curvilinear relationship between RWT and employee CD:

*H1*. The relationship between RWT and CD is curvilinear (inverse U-shaped), with the highest CD performed at an intermediate degree of RWT.

### Paradoxical impacts of KS and KH on CD

The definition of knowledge has deep historical and philosophical roots. Many philosophers in the West and East, including Plato, Aristotle, Confucius, and Laozi, have put forward various conceptions covering a wide range of doxastic notions, such as belief, ethics, esthetics, and intellectual virtues (Chappell, 2012; Chin et al., 2022). Notwithstanding the numerous epistemological debates about the exact definition of knowledge and conceptual boundaries between information and knowledge in philosophical studies (Zahra et al., 2020), many scholars have extended the connotations of knowledge from Plato’s Theaetetus and have defined knowledge as “justified true belief” (Nonaka, 1994; Wang and Chin, 2020).

Built on this view, Polanyi’s (1966) theory of tacit knowing proposes that it forms the central and indispensable element of all knowledge. This implies that tacit and explicit knowing are interdependent elements of a whole entity (Wang and Chin, 2020), in which the explicit dimension of knowledge can be easily codified and transferred through language, while the tacit dimension is deeply rooted in an individual’s mind, experience, and actions, which are hard to formalize and transmit. This theory distinguishes knowledge from information because it suggests that knowledge is derived from information but must be processed and justified by human reflection, enlightenment, and experience. Echoing this line of thought, recent research further claims that knowledge in practical utility includes information and know-how, in which it seems too trivial to distinguish knowledge from information because the two terms can be used interchangeably in KM-related studies (Chin et al., 2021b; Gagné et al., 2019; Wang and Noe, 2010; Zahedi et al., 2016). Taking the above arguments together, we also deem tacit–explicit dialectics to be the dynamic kernel of knowledge and regard *knowledge* as a fluid mix of information processed by individuals encompassing ideas, experience, values, expertise, and the like.

The present paper uses the post-COVID career landscape as the research setting in which WFA embodies a novel, contemporary form of working that entails highly flexible yet sophisticated job arrangements. However, as indicated by the Microsoft case, the WFA structure may largely complicate the flow and exchange of knowledge among employees in numerous hybrid working modes involving remote and on-site job designs. According to the Microsoft survey results (Bloom, 2014; Choudhury et al., 2019), some employees praised the timeliness and convenience of sharing information simultaneously with numerous colleagues and multitasking in the WFA setting. However, some complained that, during the brainstorming process, some team members turned their cameras off, which could conceal their immediate thoughts and make effective communication difficult. When discussing critical topics with online coworkers, it seemed harder for the team and organization to achieve a high level of understanding.

The paradoxical results found in the Microsoft case encourage us to examine the synergies of the key components of knowledge exchange, namely KS and KH. As the process of mutually exchanging and jointly creating knowledge among employees (Hooff and Ridder, 2004), KS constitutes the most critical and fundamental part of knowledge exchange (Papa et al., 2020). Research shows that KS often exerts a synergistic, cooperation-enhancing impact on positive individual outcomes, such as commitment (Cabrera and Cabrera, 2002), and on organizational performance, such as innovation (Papa et al., 2018).

As an intentional attempt by individuals to conceal or withhold knowledge solicited by coworkers (Connelly et al., 2012), KH has been widely recognized as a vital, complementary approach to deciphering KS through the understanding of not sharing (Gagné et al., 2019). According to self-determination theory, KS and KH are promoted by different intrinsic and extrinsic forms of motivation and are not necessarily contradictory (Connelly et al., 2012). Employees may strategically and intentionally hide the knowledge requested by their colleagues by being evasive, playing dumb, or giving an acceptable excuse (Duan et al., 2022).

Extending the discussion above with a cognitive frame of yin–yang harmonizing, we consider KS and KH to be the primary yin and yang elements of a complex knowledge exchange situation. A higher level of KH often implies greater degrees of interpersonal justice and power differentials among employees; in contrast, a higher level of KS indicates a greater degree of mutual trust and team cohesiveness (Wang and Noe, 2010). However, KH cannot be interpreted simply by the absence of KS but rather by an interdependent behavior or action that may affirm and negate the effect of KS on employee outcomes. Following this logic, in the WFA context, it is plausible to see the coexistence of KS and KH, while their interaction is expected to significantly affect employees’ CD. Thus, we hypothesize the following:

*H2*. The interaction of KS and KH is significantly related to CD.

### Paradox of RWT, KS, and KH in career development

Despite news reports that some people—particularly knowledge-creative workers—feel happier and become more productive in the WFA setting (Bloom, 2014), such a hybrid job design also suffers from inherent, built-in disadvantages because of (1) technology limitations (e.g., internet connection issues) that hinder knowledge flows within intranet or other digital-enabled platforms (Adisa et al., 2017) and (2) social isolation from the lack of face-to-face communication and sufficient organizational support (Bell et al., 2017). This implies substantial, somewhat contradictory associations between remote work and knowledge-related behaviors. As illustrated, the most salient feature of the WFA model lies in its job design encompassing different amounts of RWT, while the co-occurrence of KS and KH is unavoidable in this context. Thus, it is meaningful to examine the paradoxical interactions of RWT and KS–KH syncretism in employees’ CD.

From a yin–yang harmonizing cognition, we argue that, although the proportion of RWT in total working time pertains to the flexibility and autonomy of employees to engage in KS, it may also impose a veil of distortion on KS. This is because working remotely also allows employees to hide knowledge more easily because of the decreasing level of behavioral control by supervisors and organizations. Moreover, a large amount of RWT may engender employees’ distrust of each other because of the lack of accountability in online interactions (Adisa et al., 2017; Wood et al., 2018), which is associated with the display of KH in the WFA context, in which employees tend to withhold critical knowledge requested by colleagues with weak network ties.

The arguments above lead us to posit that, strategically, it seems imperative to maintain a harmonious balance between RWT and the syncretism of KH and KS. Based on hypotheses 1 and 2, we further hypothesize the following:

*H3*. The relationship between RWT and CD is moderated by the interaction of KS and KH.

The foregoing line of thought highlights that the ever-changing, dynamic, and perplexing interdependencies and interconnectedness of KS, KH, and RWT in the contemporary WFA context are expected to affect individuals’ career outcomes. To more clearly capture the dynamics of the complex KS–KH–RWT interactions, we further specify our previous moderating assumption. In hypothesis 1, we posit that, beyond a certain threshold, remote work barriers may stall employees’ CD. This is because when the proportion of the RWT of the total work time is extremely high or extremely low, individuals are more likely to be affected by the initial disruptions or prolonged remote work fatigue effects. With this premise in mind, we consider the detrimental nature of KH and the positive nature of KS in general conditions to crystallize the moderating mechanism of the KS–KH–RWT interaction in CD. Thus, we hypothesize the following:

*H4*. The interaction term has the strongest moderating effect when KS is at the highest level and KH is at the lowest level.

## Methodology

The aim of the present research is to investigate the effect of WFA on CD by considering the interaction of KS and KH in this relationship. To test the relationship, we adopted a quantitative survey design, which can provide a general understanding of the participants’ views and opinions in an entire population (Creswell and Plano Clark, 2018; Wakhata et al., 2022).

### Participants

This research project was initiated in late April 2021 and lasted for three months, including two time points. Because it was difficult to move between Chinese provinces amid the COVID-19 outbreak, we chose seven manufacturers that had adopted hybrid job designs of remote and on-site work located in Zhejiang Province as the sample firms. In each firm, at least one contact person from the human resources (HR) department helped distribute the online questionnaires. The questionnaires were distributed among workers in the sample firms by applying simple random sampling techniques (Mansor et al., 2021; Radwan, 2022). Before the formal surveys were administered, the research team conducted in-depth telephone interviews with several HR professionals and senior employees at the selected firms to ensure the appropriateness and clarity of the measurements and hypotheses. Their feedback confirmed that the hypothesized model was logical. To ensure the validity of this research, we selected only full-time employees as the participants.

In terms of carrying out the formal online survey, we employed a reliable online service provider in China—Sojump (https://www.wjx.cn/)—to distribute the questionnaires to participating workers via WeChat. We also provided clear guidelines for how the respondents should fill out the questionnaire and how their anonymity was protected. A WeChat account/ID was offered to answer the participants’ inquiries.

To reduce the likelihood of common method variance (CMV), we collected data at two time points to meet the conditions of the time lag research design (Shin et al., 2018). We measured RWT, KS, and KH at point 1 and career development (CD) at point 2 (three months later). As a result, usable data from a total of 364 employees (with point 1 and point 2 data) were obtained, with a response rate of 70.3%. The sample included 181 women and 183 men, with an average age of 33.5 years, of whom 64.6% were married and 35.4% were unmarried; of the participants, 19.2% were from state-owned companies, and 80.8% were from other types of companies.

### Measures

Because Chinese employees are apt to disguise their true feelings by choosing the midpoint of a scale, the participants were asked to respond on a 6-point Likert-type scale ranging from “strongly disagree” to “extremely agree” to prevent response bias (Chin, 2015).

#### Remote work time (RWT)

Referring to Golden (2006) and Wiesenfeld et al. (1999), we assessed RWT by asking the participants to report the proportion of the average workweek they spent teleworking. Higher scores indicate that the participants spent more time working remotely. The responses ranged from 0% to 100%, with a mean of 44%.

#### Knowledge sharing (KS)

Given that Chinese employees may not be able to truthfully report their behavior, we used a peer rating rather than a self-report measure (Chin, 2015). We adopted Hu et al. (2009) 11-item scale and adjusted its items to measure employees’ perceptions of their colleagues’ degree of KS. The sample items included the following: “My colleague enjoys learning and sharing knowledge through teamwork,” “My colleague will personally help other team members regardless of whether they are in need,” and “My colleague is willing to help other team members” (Cronbach’s *α* = 0.942).

#### Knowledge hiding (KH)

As mentioned above, we also adjusted Connelly et al.’s (2012) scale to measure employees’ perceptions of the degree of their colleagues’ degree of KH. Sample items included the following: “My colleague said that he/she did not know, even though he/she did,” and “My colleague said that he/she would not answer my questions” (Cronbach’s *α* = 0.935).

#### Career development (CD)

Because of the lack of mature scales for CD, we referred to Yoon et al.’s (2021) suggestion that deems individuals’ capacity to exercise control over their career as the core component of CD as our method to analyze CD with a proximate measure, namely the four-item career control indicators proposed by Akkermans et al. (2013). Sample items included the following: “I am able to set goals for myself that I want to achieve in my career” (Cronbach’s *α* = 0.911).

#### Control variables

We controlled for age (in years), gender (0 = male, 1 = female), marriage (0 = not married, 1 = married), type of firm (0 = state-owned, 1 = other), and firm size (1 = fewer than 80 employees, 2 = 81–500 employees, 3 = 501–1000 employees, 4 = 1001–2000 employees, 5 = more than 2000 employees).

## Results

### Reliability and validity

The values of Cronbach’s *α* for all measures were above 0.70, indicating acceptable reliability. The values of construct reliability (CR) and average variance extracted (AVE) were above the acceptable values of 0.7 and 0.5, respectively (see Table 1). We then assessed the discriminant validity using a series of confirmatory factor analyses (CFAs). As shown in Table 2, the assumed full model displayed the best fit to our data, confirming the nomological validity (*χ*^2^_*n*=364_ = 237.337, df = 71, *χ*^2^/df = 3.342, *P* < 0.001, CFI = 0.960, TLI = 0.948, RMSEA = 0.080, SRMR = 0.045). According to Podsakoff et al. (2012), the goodness-of-fit indexes above and the values of the interconstruct correlations (see Table 3) were within acceptable limits; thus, the convergent and discriminant validity of the scales were verified.

**Table 1:** Factor loadings of perceptual scales.

Constructs	Measurement items	Factor loadings	AVE	CR
KS	My colleague respect others’ impression that he/she is willing to assist people	0.832	0.646	0.948
	In a team setting, my colleague is willing to share knowledge to pay back others that have assisted him/her	0.832		
	My colleague wants to become a person with professional knowledge in the eyes of others	0.825		
	My colleague believes that knowledge sharing among teams can help establish his/her image as an expert	0.818		
	Helping the team address work problems will let my colleague feel happy and satisfied	0.811		
	My colleague believes that members should help each other to foster knowledge sharing	0.804		
	My colleague enjoys learning and sharing knowledge through teamwork	0.784		
	My colleague will personally help other team members, regardless of whether they are in need	0.781		
	My colleague is willing to help other team members	0.778		
	My colleague is willing to use his/her spare time to help other team members	0.775		
	My colleague is eager to exchange knowledge without asking for anything in return	0.652		
KH	Said that he/she did not know, even though he/she did	0.934	0.763	0.950
	Pretended he/she did not know what I was talking about	0.918		
	Told me that he/she would help me out later but stalled as much as possible	0.900		
	Agreed to help me but instead gave me information different from what I wanted	0.898		
	Told me that his/her boss would not let anyone share this knowledge	0.862		
	Said that he/she would not answer my questions	0.713		
CD	I can create a layout for what I want to achieve in my career	0.915	0.745	0.921
	I can set goals for myself that I want to achieve in my career	0.889		
	I can make clear career plans	0.851		
	I know what I want to have achieved in my career a year from now	0.793

**Table 2:** CFA results.

Models	*χ*^2^	DF	χ2/DF	RMSEA	CFI	TLI	SRMR
Full Model	237.337	71	3.342	0.080	0.960	0.948	0.045
3-Factor Model	931.449	74	12.587	0.178	0.792	0.744	0.120
2-Factor Model	2019.500	76	26.572	0.265	0.528	0.434	0.247
1-Factor Model	2912.517	77	37.825	0.318	0.311	0.186	0.279

3-Factor Model, KS + CD&KH&RWT; 2-Factor Model, KS + CD + KH&RWT; 1-Factor Model, KS + CD + KH + RWT.

**Table 3:** Descriptive statistics and correlations.

Variable	Mean	S.D.	RWT	KS	KH	CD
RWT	0.44	0.29	–			
HS	3.16	0.83	0.023*	**0.64**		
KH	2.81	1.78	−0.109*	0.088*	**0.76**	
CD	4.30	0.99	0.369***	0.537***	−0.047*	**0.74**

*N* = 364. The values of the square roots of AVE (average variance extracted) are shown on the diagonal in bold.

***p < 0.001, *p < 0.050.

### Common method variance (CMV)

We conducted the CFA marker method (Podsakoff et al., 2012) to test CMV. Compared with the original full model (*χ*^2^_*n*=364_ = 237.337, df = 71, *χ*^2^/df = 3.342, *P* < 0.001, CFI = 0.960, TLI = 0.948, RMSEA = 0.080, SRMR = 0.045), the new model with the latent CMV factor still fit the data well (*χ*^2^_*n*=364_ = 252.125, df = 84, *χ*^2^/df = 3.001, *P* < 0.001, CFI = 0.962, TLI = 0.952, RMSEA = 0.074, SRMR = 0.043) and did not show any significant differences (Δ*χ*^2^ = 14.778, *P* > 0.050). Hence, CMV is unlikely to be a major threat.

### Hypotheses test

To test the proposed hypotheses, we conducted a moderated hierarchical regression analysis that can estimate the relationship among independent, dependent, and moderating variables (Samuel, 2021). Moderated hierarchical regression analysis is regarded as a relatively conservative method for verifying the interaction effects because the interaction term is examined for significance after all lower-order effects (Jaccard et al., 1990; Wang et al., 2020). To avoid multicollinearity, we mean-centered all variables first (Cohen et al., 2003). We then added the independent variable, the two-way interaction terms, and the three-way interaction terms sequentially into the regression (see Table 4).

**Table 4:** Results of the regression analysis.

Variables	Model 1	Model 2	Model 3	Model 4	Model 5	Model 6	Model 7	Model 8	Model 9	Model 10
Age	0.021^***^	0.019^**^	0.018^***^	0.09	0.010^†^	0.009^†^	0.010^†^	0.010^†^	0.009^†^	0.009^†^
Gender	0.070^*^	0.031	0.033	0.033	0.034	0.013	0.023	0.024	0.020	0.016
Marriage	−0.020	−0.031	−0.062^*^	0.017	0.007	−0.026	−0.053	−0.054	−0.045	−0.064^*^
Scale	−0.014	−0.017	−0.034	−0.008	−0.008	−0.028^*^	−0.029^*^	−0.028	−0.029	−0.035
Property	0.114^*^	0.072	0.059	0.071	0.071	0.032^*^	0.030^*^	0.030^*^	0.027^*^	0.027^*^
RWT		1.918^***^	2.883^***^			2.143^***^	2.091^***^	2.077^***^	2.176^***^	2.235^***^
RWT^2^			−5.599^***^			−4.169^***^	−3.780^***^	−3.623^***^	−3.777^***^	−3.567^***^
KS				0.647^***^	0.618^***^	0.553^***^	0.545^***^	0.542^***^	0.554^***^	0.502^***^
KH				−0.475^*^	−0.498^**^	0.440	−0.346	−0.344	−0.376^†^	−0.393
KS × KH					0.080^*^	0.006	0.013	0.006	0.008	0.097
RWT × KS							0.141	−0.183	0.125	−0.187
RWT × KH							0.399	0.428	0.299	0.312
RWT × KS × KH								−0.104	−0.135	0.041
RWT^2^ × KS									−0.196	0.944
RWT^2^ × KH									0.843	1.013
RWT^2^ × KS × KH										−1.421^**^
*R*^2^	0.041^*^	0.167^**^	0.253^***^	0.316^***^	0.323^***^	0.405^***^	0.410^***^	0.413^***^	0.424^***^	0.430^***^

*N* = 364.

***p < 0.001, **p < 0.010, *p < 0.050, ^†^*p* < 0.100.

**Fig. 1:**
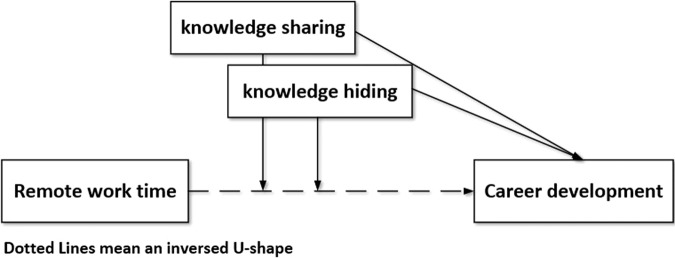
The relationships among the variables. Remote work time is independent variable, career development is dependent variable, knowledge sharing and knowledge hiding are moderating variables.

As shown in Model 2, RWT was positively related to CD (*β* = 1.918, *P* < 0.001). In Model 3, the square term of RWT was added. The results revealed that the square term of RWT had a significantly negative relationship with CD (*β* = –5.599, *P* < 0.001), indicating that RWT had an inverse U-shaped relationship with CD; hypothesis 1 was supported. Model 4 indicated that KS was positively related to CD (*β* = 0.647, *P* < 0.01), while KH was negatively related to CD (*β* = −0.475, *P* < 0.05). Model 5 added the interaction term of KS and KH, and the results showed that the interaction term of KS and KH was positively related to CD (*β* = 0.080, *P* < 0.05); hypothesis 2 was supported (Fig. 1).

To verify the relationship between the three-way interaction terms of RWT, KS, and KH in the employees’ CD, Model 6 through Model 10 conducted hierarchical regressions. According to Model 10, the U-shaped relationship between RWT and CD was moderated by the joint effect of KS and KH (*β* = –1.421, *P* < 0.001); hypothesis 3 was supported.

**Fig. 2:**
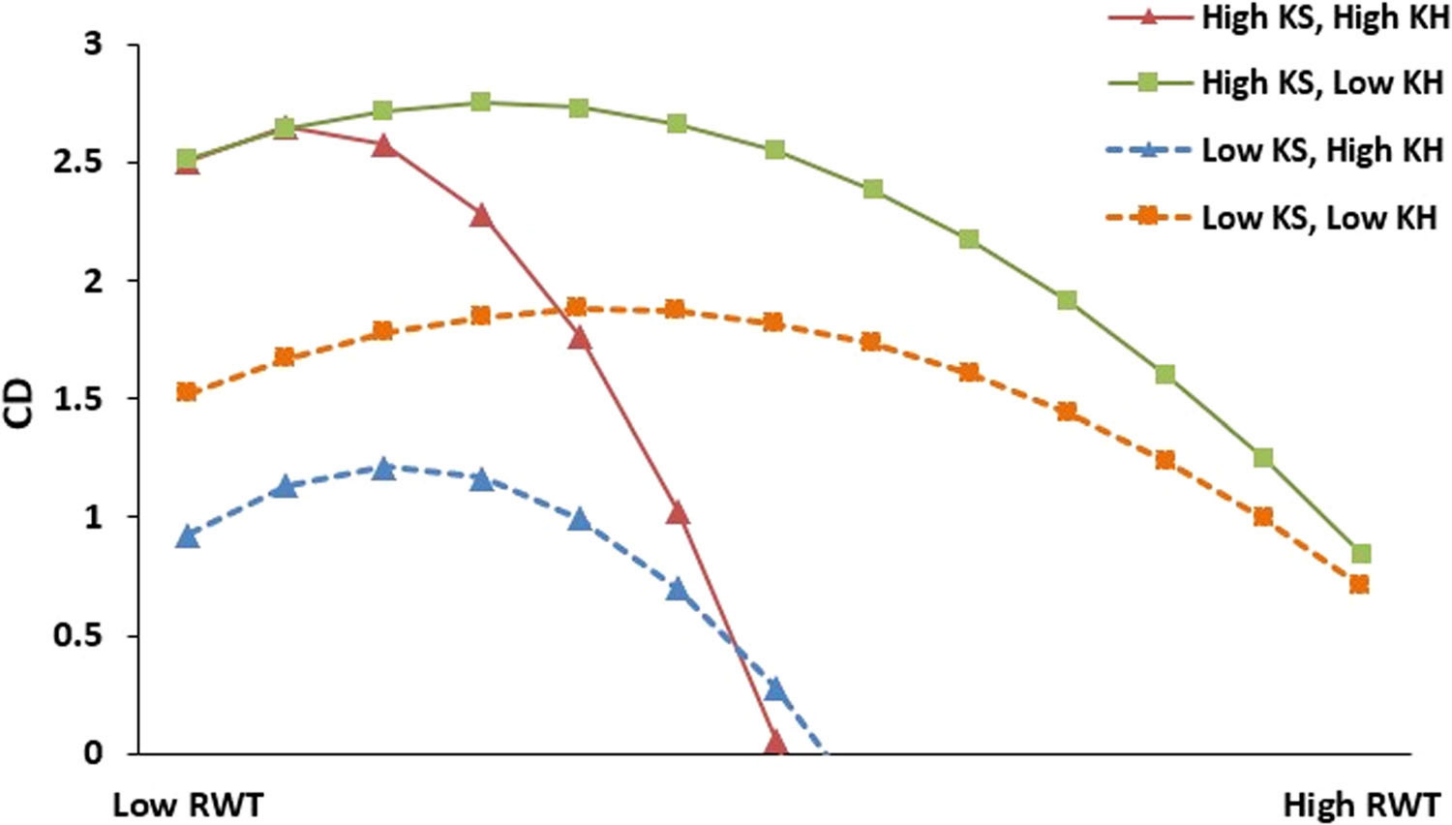
Three-way interaction plot. Description: Three-way interaction plot reflects the moderating effects of knowledge sahring (KS) and knowledge hiding (KH) on the relationship between remote work time (RWT) and career dvelopment (CD).

In addition, referring to Dawson (2014), we also plotted the three interactions in Fig. 2 (RWT^2^ × KS × KH); the interaction term of KS and KH has the strongest moderating effect on the relationship between RWT and CD when KS is at the highest level and KH at the lowest level. The plot further confirmed hypothesis 4.

## Discussion

The empirical analysis showed that all four hypotheses were fully supported. A curvilinear (inverted U-shaped) relationship exists between RWT and CD, where the highest CD is found at the intermediate degree of RWT. The interaction of KS and KH is significantly related to CD. Moreover, the relationship between RWT and CD is moderated by the interaction of KS and KH; thus, the interacting term of KS and KH has the strongest positive moderating effect on the relationship between RWT and CD when KS is at the highest level and KH is at the lowest level. In terms of the control variables, the results suggest a positive association between employees’ age (older), gender (female), and type of firm (state-owned company), as well as a negative association between employees’ marriage status (nonmarried) and their CD. Overall, the current research enriches our understanding of a new form of job design—WFA—by linking it to the intersection of the career and KM domains through an unorthodox paradoxical lens. We elaborate on the main contributions below.

### Theoretical contributions

First, based on the cognitive frame of yin–yang harmonizing, we contribute to the HRM literature by examining the nonlinear effect of remote working and symbiotic impact of KS and KH on CD. The results highlight the emergence and existence of a variety of micro-level paradoxes in terms of developing careers during the COVID-19 pandemic. Specifically, the inverted U-shaped relationship between RWT and CD partly echoes the autonomy control view (Bathini and Kandathil, 2019; Putnam et al., 2014) that remote working engenders not only greater work availability, but also work intensification because of the less visible, unobtrusive control mechanism residing in the social norm of being an ideal worker by working harder and longer. Meanwhile, such a nonlinear, inverted U-shaped mechanism may be especially prominent among employees whose job duties require a lot of coordination with coworkers because they may feel anxious and depressed when they perceive that they are receiving little support from colleagues and supervisors. In conclusion, we provide novel insights into a more comprehensive understanding of HRM in a perplexing WFA context.

Second, the current research adds value to the KM literature by examining the interconnectedness of KS and KH and their interacting effects on HRM-related outcomes. Despite obvious antagonism (KS positive and desirable and KH negative and undesirable), KS and KH are actually not opposing notions, but two independent yet interconnected concepts in social interactions (Connelly et al., 2012; Garcia et al., 2020). The WFA model presents a modern, intricate, yet smart infrastructure for employees to engage in knowledge exchange because it engenders a greater diversity of communication modes, connection patterns, and social relationships for mobile employees and teleworkers. Although it is plausible to see KS and KH occur synchronously in such a context, limited empirical research has articulated the synergistic interaction of KS and KH in HR practice in the organizational context. The present research, which has examined the interacting mechanisms of KS and KH in career consequences, can thus be seen as an exciting new step, exposing the need to incorporate unconventional, cross-disciplinary perspectives to interpret relevant issues.

Related to the points above, we have adopted the paradoxical cognition of yin–yang harmonizing to investigate the synergistic interaction of KS and KH, thus advancing the traditional binary analysis of on-site and remote works to an unorthodox, contemporary understanding of flexible and dynamic work arrangements in the digital economy. To a certain extent, these initial findings can inspire scholars to dig deeper into this very important yet underexplored area of inquiry in the “new normal.”

### Managerial implications

The findings of the present study offer valuable implications for global managers and workers in coping with bewildering work relationships and growing career challenges in a digitized and volatile workplace.

First, the present study highlights that remote work acts as a nonlinear relationship with respect to the CD of employees in need of support and cooperation. This means that managers should build a hybrid work community based on shared values, both physically and digitally, to foster support and cohesion within the team. Targeting employees who do not need access to hardware that is difficult to move or whose job duties have little or no coordination with coworkers, companies could eye policies that balance the amount of time employees work at home and in the office during the week, which may be more ideal for companies and employees than full time in the office or full time at home.

Second, the present study confirmed that flexibility in places and times of work increases the personal satisfaction of employees who can organize and manage their day according to their needs. Therefore, even if there is indirect environmental interference while employees work remotely, work flexibility and ICT can still favor the exchange of knowledge through the interaction between KS and KH in proportion to the time spent working remotely. In accordance with the view of autonomy control (Bathini and Kandathil, 2019; Putnam et al., 2014), the study results partly suggest that giving employees greater autonomy in the choice of WFA permanently or temporarily makes them feel more in control of their careers.

However, the study also revealed that WFA is likely to reduce KS and increase KH under conditions of low physical embodiment. Therefore, the results suggest that managers should take various measures to enhance the connection and cohesion of employees and teams, as well as to deal with the problem of “information islands” in the WFA setting. For example, instead of simple voice or chat messages, employees should be encouraged to connect with their workmates in online meetings with their cameras on because such face-to-face meetings increase physical embodiment and build trust and a sense of teamwork. Furthermore, managers should adopt dynamic organizational strategies capable of spreading the work culture based on the results. Several studies have examined the propensity of distance workers to be promoted, despite the career development they have provided to the organization because they are believed to be underutilized. This implies that managers should adopt reward systems based on the recognition of the performance achieved by remote workers.

### Limitations and future research

Despite the originality of this contribution to the HRM literature, the present study has some limitations that pave the way for new avenues of research. First, the current research has identified the paradoxical mechanisms of RWT–KS–KH interactions in CD in a novel, ICT-enabled WFA context. However, given that knowledge exchange is context specific (Chin et al., 2021b; Papa et al., 2020), future research could be extended to other contexts and further capture the gray areas of KS and KH because KS may have a dark side while KH has some positive effects in exceptional circumstances. Second, the sample was composed of companies located in China’s Zhejiang Province that have adopted hybrid jobs and remote job projects. In this regard, the analysis could be extended to other countries, verifying the results and carrying out comparative context analyses. Another limitation is the sector of the selected companies that operate in the manufacturing industry. Future research could conduct empirical analyses in other areas, highlighting the similarities and differences in approaches to WFA and CD. Finally, the present study has conducted an analysis at the micro level but offers interesting insights from a relational and sociological point of view. Therefore, future studies could extend the analysis through a cross-cutting approach that includes social and psychological variables.

## Conclusion

Distinct from typical dehumanized remote working conditions, the modern ICT-enabled WFA context transcends the traditional spatiotemporal boundaries of work structures and characterizes *a multitiered, smart infrastructure of employee knowledge exchange*, where KS and KH also go beyond the original work and job premises and time. However, the impact on HRM-related outcomes remains somewhat puzzling. For instance, as shown by some empirical surveys, employees hailed this shift as surprisingly productive in the beginning, but after a while, they became frustrated with the difficulties of converging on the meaning of complex information through virtual meetings and the lack of division between their work and private lives. Thus, we believe that, over time, coupled with the rapid advancement of ICT, the WFA model may continuously evolve into more configurations in the contemporary career landscape. It entails the building of more sophisticated knowledge networks that allow for kaleidoscopic knowledge exchange activities and polychronic knowledge creation among employees with diverse forms of employment.

## Data Availability

The datasets generated during and/or analyzed during the current study are not publicly available due to ongoing research and analysis, but are available from the corresponding author on reasonable request.

## Footnotes

1 https://www.silicon.co.uk/workspace/microsoft-permanent-remote-work-348265
